# Diagnostic delay in patients from the International Map of Axial Spondyloarthritis: geographic, sociodemographic and disease-related factors

**DOI:** 10.1093/rheumatology/keae521

**Published:** 2024-09-25

**Authors:** Denis Poddubnyy, Marco Garrido-Cumbrera, Fernando Sommerfleck, Victoria Navarro-Compán, Christine Bundy, Souzi Makri, José Correa-Fernández, Shashank Akerkar, Jo Davies, Elie Karam

**Affiliations:** Department of Rheumatology, Charité - Universitätsmedizin Berlin, Berlin, Germany; Department of Rheumatology, German Rheumatology Research Centre, Berlin, Germany; Health & Territory Research (HTR), Universidad de Sevilla, Seville, Spain; Spanish Federation of Spondyloarthritis Associations (CEADE), Madrid, Spain; Department of Rheumatology, Sanatorio Julio Mendez, Buenos Aires, Argentina; Department of Rheumatology, IdiPaz, Hospital Universitario La Paz, Madrid, Spain; Department of Rheumatology, Cardiff University, Cardiff, UK; Cyprus League for People with Rheumatism (CYLPER), Nicosia, Cyprus; Health & Territory Research (HTR), Universidad de Sevilla, Seville, Spain; Department of Rheumatology, Mumbai Arthritis Clinic, Mumbai, India; Axial Spondyloarthritis International Federation (ASIF), London, UK; Canadian Spondylitis Association (CSA), Toronto, Canada

**Keywords:** axial spondyloarthritis, diagnostic delay, geographic, patient-reported outcomes

## Abstract

**Objectives:**

To assess diagnostic delay and its associated factors globally, in a large sample of patients included in the International Map of Axial Spondyloarthritis (IMAS).

**Methods:**

IMAS is a cross-sectional online survey (2017–22) of 5557 axial spondyloarthritis (axSpA) patients from 27 countries. Diagnostic delay was calculated as the difference between age at diagnosis and age at first symptom onset reported by patients. Associations between diagnostic delay and regions, sociodemographic characteristics and disease-related factors were explored through univariable and multivariable linear regression analysis.

**Results:**

Data from 5327 patients who reported data on diagnostic delay in IMAS survey were analysed: 3294 were from Europe, 752 from North America, 590 from Asia, 545 from Latin America and 146 from Africa. Overall, patients reported a mean diagnostic delay of 7.4 years (median: 4.0) since symptom onset, with substantial variation across regions; the highest delay was in South Africa and the lowest in Asia. The variables associated with longer diagnostic delay in the final multivariable regression model were: younger age at symptom onset (b = –0.100), female gender (b = 2.274), being diagnosed by a rheumatologist (b = 1.163), greater number of heathcare professionals (HCPs) seen before diagnosis (b = 1.033) and history of uveitis (b = 1.286).

**Conclusion:**

In this global sample of axSpA patients the mean diagnostic delay was 7.4 years, and showed significant differences across regions. Younger age at symptom onset, female gender, diagnosis made by a rheumatologist, greater number of HCPs seen before diagnosis and history of uveitis were the parameters associated with a longer diagnostic delay in axSpA patients.

Rheumatology key messages

- The diagnostic delay for patients was longer than 7 years, reaching almost 11 years in South Africa.
- Greater number of healthcare professionals seen before diagnosis was associated with longer diagnostic delay.
- Patients with longer diagnostic delay were associated with history of uveitis.

## Introduction

Axial SpA (axSpA) is a chronic inflammatory disease characterized by involvement of the axial skeleton (sacroiliac joints and spine) [1], peripheral joint involvement [2], the presence of enthesitis and dactylitis [3, 4], typical extra-musculoskeletal manifestations such as uveitis, psoriasis and IBD [5], and association with the HLA-B27 antigen [6].

Despite knowledge of these features, axSpA patients experience diagnostic delay. A recent meta-analysis showed a diagnostic delay of 6.7 years, being higher in Europe than in the West Pacific or Eastern Mediterranean [7]. Several studies have shown that longer diagnostic delay is associated with worse outcomes, including greater disease activity, worse treatment response and higher level of work disability [8–10].

Previously, we reported on axSpA diagnostic delay in Europe. In this study, longer diagnostic delay was associated with younger age at symptom onset, female sex and with greater number of healthcare professionals (HCPs) seen before diagnosis [11]. The aim of the current study was to assess diagnostic delay and factors associated with it in a large sample of patients globally, comprising in the International Map of Axial Spondyloarthritis (IMAS).

## Methods

### Design and survey development

The IMAS initiative is a research collaboration between the Axial Spondyloarthritis International Federation (ASIF), the Health and Territory Research (HTR) group of the University of Seville and Novartis Pharma AG, together with a scientific committee composed of axSpA patient research partners, rheumatologists, psychologists and health researchers. IMAS involves 27 countries worldwide: Argentina, Austria, Belgium, Brazil, Canada, Colombia, Costa Rica, France, Germany, India, Italy, Korea, Lithuania, Mexico, the Netherlands, Norway, Philippines, Russia, Slovenia, South Africa, Spain, Sweden, Switzerland, Taiwan, Turkey, the UK and the USA. The IMAS questionnaire was originally developed in Spanish and subsequently translated into the main language of each of the 27 participating IMAS countries [12]. The questionnaire included over 120 items and was administered through an online survey platform managed by Ipsos. More information of design and dissemination of the survey has been already described in the seminal manuscripts at European and International level [12, 13].

### Participants and recruitment

**Figure 1.**
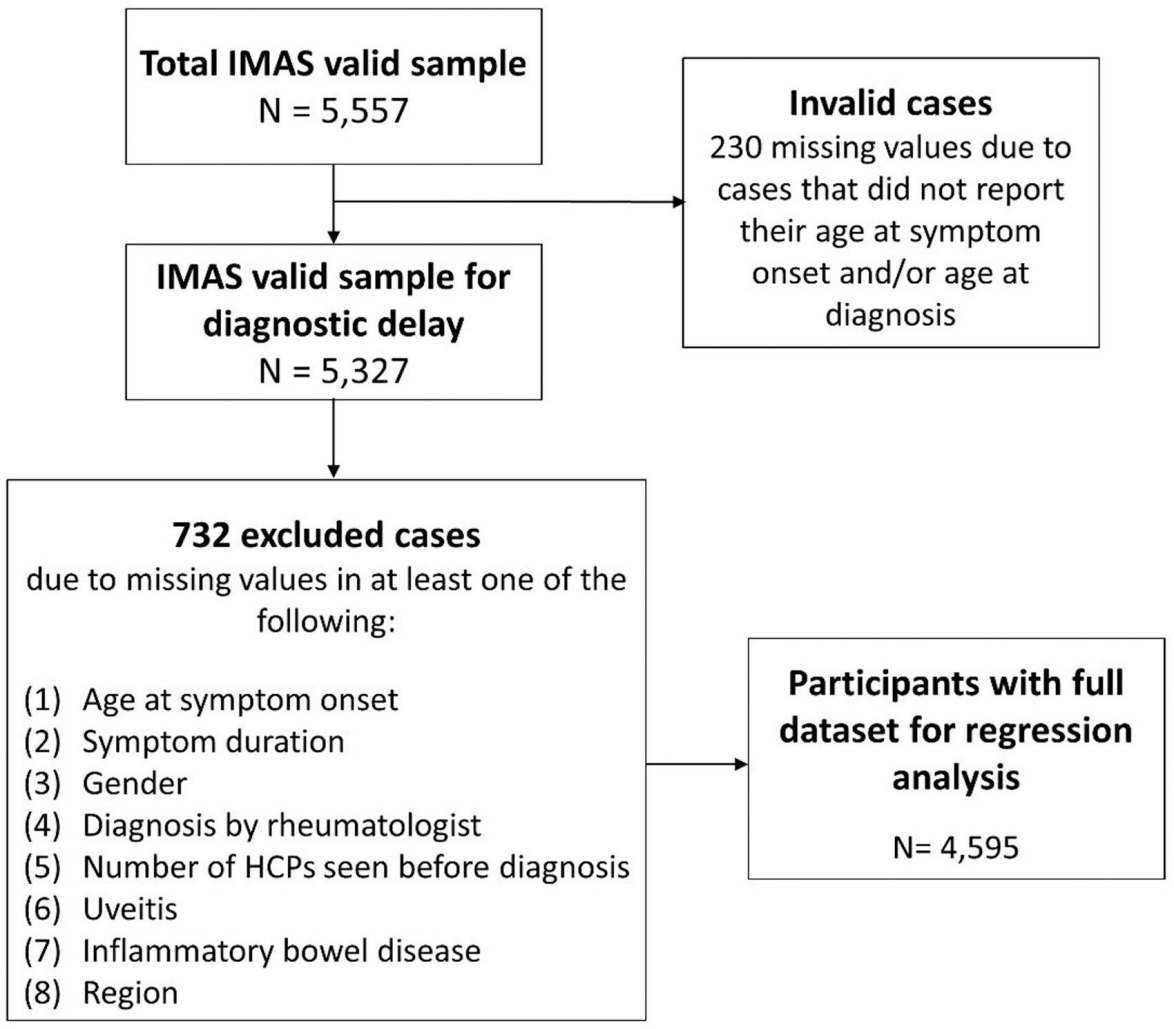
Flowchart of sample selection

Unselected patients were recruited on a voluntary basis from an internal Ipsos panel and local patient organizations between 2017 and 2022. The questionnaire was administered via an online platform for survey data collection. Coordination of the patient survey and data collection was led by Ipsos S.A. (Fig. 1). Age at least 18 years, residents of one of the specific selected countries and self-reported diagnosis of axSpA [either AS—also referred to as radiographic (r) axSpA or non-radiographic (nr) axSpA] made by a rheumatologist or another HCP were the selection criteria for IMAS participants.

### Collected data

The description of sociodemographic, diagnosis characteristics and disease extra-musculoskeletal manifestations used in the present analysis are described in Supplementary Table S1 (available at *Rheumatology* online). Diagnostic delay was calculated as the difference between the following two items from the IMAS survey: ‘Age at first symptom onset (pain, inflammation, stiffness) associated with Spondylitis/Spondyloarthritis’ and ‘Age at which you were diagnosed with Spondylitis/Spondyloarthritis’.

### Statistically analysis

Mann–Whitney test was used to compare the diagnostic delay between subgroups defined by variables with two categories: gender (male, female), diagnosed by a rheumatologist (yes, no), HLA-B27 (positive, negative), presence of uveitis (yes, no), presence of psoriasis (yes, no) and presence of IBD (yes, no). The Kruskal–Wallis test was used to evaluate the differences in diagnostic delay between subgroups defined by variables with more than two categories: age at symptom onset (≤18, 19–34, 35– 51, 52–70 years), educational level (no schooling completed, primary school, high school, university) and number of HCPs seen before diagnosis (0, 1–2, 3 or more).

Univariable and multivariable linear regression analysis was used to evaluate the relationship between diagnostic delay and candidate variables (age at symptom onset, gender, being diagnosed by rheumatologist, number of HCPs seen before diagnosis, uveitis, IBD and region). The multivariable model was additionally adjusted for symptom duration. The factor region was introduced as a dummy variable taking Europe (region with the largest sample size) as a reference. The regression coefficients (b) and corresponding 95% CIs were reported. SPSS 26.0 version was used to carry out the analysis.

The present manuscript does not contain any studies with animal subjects and Institutional Review Board approval was not necessary. All participants were asked to provide explicit opt-in consent prior to participating in the IMAS survey. Furthermore, the participants’ data were anonymized and did not contain confidential, personal or subject-identifying information. Ethical aspects related to data extracted from patients and their treatment were in accordance with the Declaration of Helsinki.

## Results

**Map 1.**
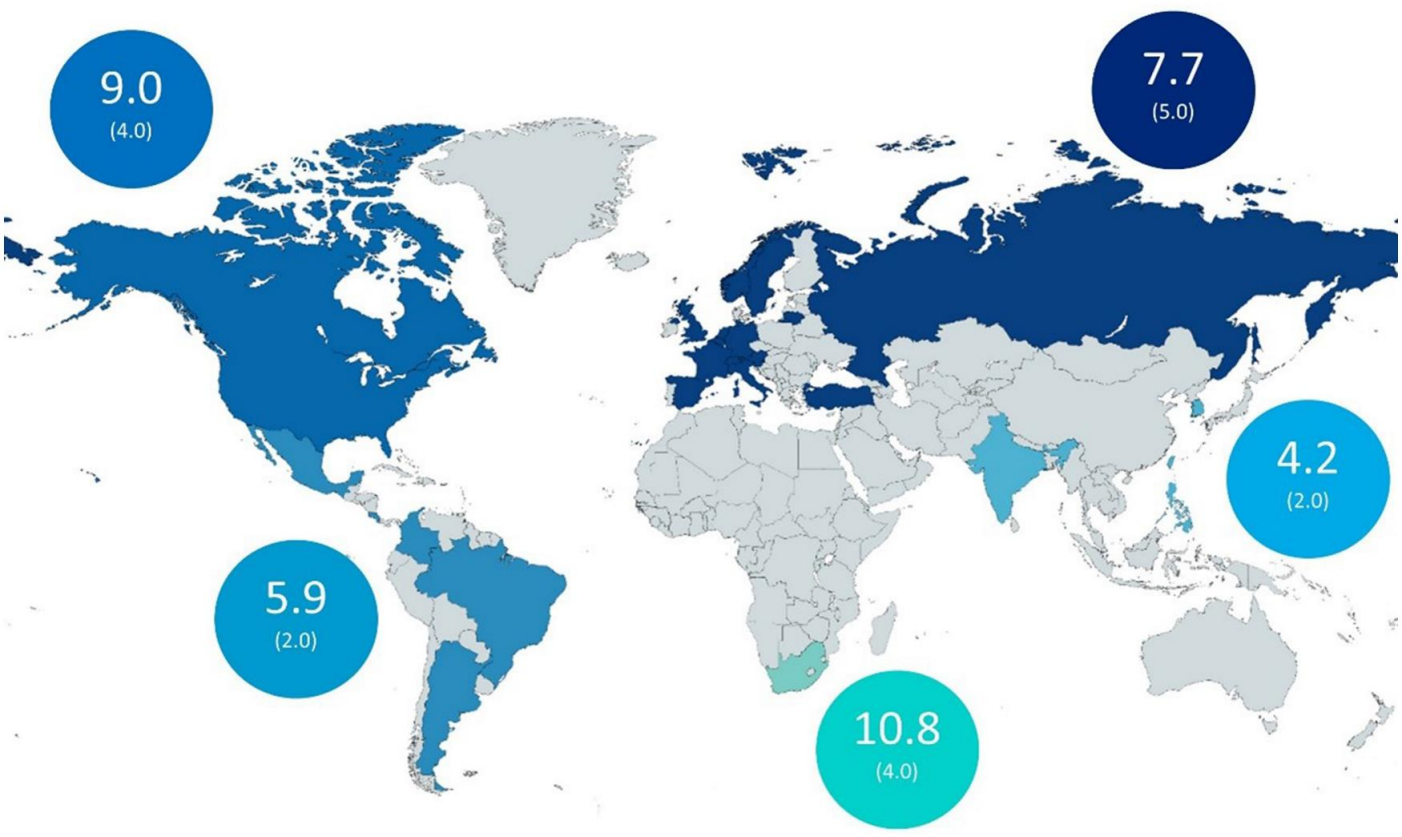
Mean and median diagnostic delay by region (*N* = 5327). Data shown in the circles refer to the mean (median) values in years

Of 5557 IMAS participants, 5327 were included as they provided information on both age at symptom onset and age at diagnosis. Overall, mean (±s.d.) age at symptom onset was 26.8 years (±11.3), mean age at diagnosis was 34.2 years (±11.4), resulting a mean of diagnostic delay of 7.4 years (±9.0; Table 1). The region with the longest diagnostic delay was South Africa (10.8 ± 10.6), followed by North America (9.0 ± 11.0), Europe (7.7 ± 8.8), Latin America (5.9 ± 8.6) and Asia (4.2 ± 5.4; Map 1).

**Table 1.** Overall and regional baseline characteristics of participants included in the diagnostic delay analysis

Variables	Mean ± s.d. or *n* (%)					
	Total	Europe	North America	Latin America	Asia	South Africa
Gender: female	3080 (55.4)	2049 (58.7)	479 (62.3)	307 (56.0)	125 (20.8)	120 (82.2)
Education level: university	2569 (46.2)	1667 (47.7)	445 (57.8)	221 (40.3)	182 (30.3)	54 (37.0)
Age at symptom onset	26.8 ± 11.3	26.2 ± 10.8	26.4 ± 12.1	30.5 ± 12.9	26.9 ± 10.7	26.7 ± 11.4
Age at diagnosis	34.2 ± 11.4	33.9 ± 11.0	35.3 ± 12.8	36.5 ± 11.4	31.0 ± 10.4	37.5 ± 10.8
Diagnostic delay	7.4 ± 9.0	7.7 ± 8.8	9.0 ± 11.0	5.9 ± 8.6	4.2 ± 5.4	10.8 ± 10.6
Symptom duration	17.1 ± 13.3	18.4 ± 13.6	18.0 ± 14.4	13.7 ± 11.2	11.6 ± 9.3	18.0 ± 12.7
Diagnosed by rheumatologist	3842 (72.1)	2499 (76.5)	511 (66.4)	407 (74.3)	323 (53.8)	102 (70.8)
No. of HCPs seen before diagnosis	2.3 ± 1.3	2.4 ± 1.4	2.6 ± 1.5	2.2 ± 1.2	1.6 ± 0.9	2.0 ± 1.1
HLA-B27: positive	2464 (71.1)	1559 (70.6)	340 (73.0)	228 (65.1)	254 (78.2)	83 (72.2)
Uveitis	1171 (23.2)	670 (21.1)	215 (30.8)	121 (24.8)	131 (23.8)	35 (25.2)
Psoriasis	461 (20.4)	265 (23.0)	46 (26.3)	109 (22.9)	25 (7.9)	16 (11.9)
IBD	724 (14.0)	391 (11.9)	128 (18.3)	84 (17.5)	88 (16.1)	33 (24.1)

HCP: healthcare professional.

Patients reported a symptom duration of 17.1 years, 55.4% were female, almost half had university education, 72.1% were diagnosed by rheumatologist, with more than two visits to HCPs for diagnosis, 71.1% had positive HLA-B27 test, 23.2% with uveitis, 20.4% with psoriasis and 14.0% with IBD (Table 1).

In the bivariate analysis, longer diagnostic delay was more frequent in those younger at symptom onset, in females, those diagnosed by a rheumatologist, those who saw more HCPs before diagnosis, and those who report ever uveitis and IBD (all *P*-values <0.05; Table 2). Bivariate analyses between independent variables and diagnostic delay for each region are available in Supplementary Table S2 (available at *Rheumatology* online).

**Table 2.** Bivariate analysis between sociodemographic and disease-related variables and diagnostic delay in the total sample

Variable		Diagnostic delay, mean ± s.d. or r correlation	*P*-value
Age at symptom onset (years)	≤18	12.8 ± 11.6	**<0.001**
	19–34	6.7 ± 7.7	
	35–51	3.5 ± 4.6	
	52–70	1.7 ± 2.6	
Gender	Male	6.1 ± 7.8	**<0.001**
	Female	8.5 ± 9.7	
Education level	No schooling completed	9.0 ± 11.0	0.468
	Primary school	7.4 ± 8.7	
	High school	7.5 ± 8.9	
	University	7.3 ± 9.0	
Diagnosed by rheumatologist	Yes	8.1 ± 9.3	**<0.001**
	No	5.7 ± 7.9	
No. of HCPs seen before diagnosis	0	5.0 ± 7.1	**<0.001**
	1–2	5.8 ± 8.0	
	3 or more	9.7 ± 9.7	
HLA-B27	Positive	8.2 ± 8.8	0.988
	Negative	8.5 ± 9.6	
Uveitis	Yes	8.7 ± 9.3	**<0.001**
	No	7.1 ± 8.7	
Psoriasis	Yes	8.5 ± 10.6	0.081
	No	7.0 ± 8.6	
IBD	Yes	8.2 ± 9.5	**0.028**
	No	7.4 ± 8.8	

*P*-values <0.05 considered statistically significant are represented in bold text. HCP: healthcare professional.

In the multivariable analysis, a longer diagnostic delay was associated with younger age at symptom onset (b = –0.100, 95% CI –0.120, –0.080), female gender (b = 2.274, 95% CI 1.860, 2.687), diagnosis by a rheumatologist (b = 1.163, 95% CI 0.710, 1.615), higher number of HCPs seen before diagnosis (b = 1.033, 95% CI 0.877, 1.189) and presence of uveitis (b = 1.286, 95% CI 0.808, 1.764). Furthermore, South Africa (b = 3.356, 95% CI 2.170, 4.541), North America (b = 1.470, 95% CI 0.902, 2.039) and Asia (b = 1.003, 95% CI 0.334, 1.673) were associated with longer diagnostic delay using Europe as a reference (Table 3).

**Table 3.** Univariable and multivariable linear regression analysis of the association between diagnostic delay and independent variables in patients with axial SpA (*N* = 4595)

Variables	Ref.	Univariable analysis		Multivariable analysis^a^	
		b	95% CI	b	95% CI
Female gender	Male	**2.324**	**1.843, 2.804**	**2.274**	**1.860, 2.687**
Age at symptom onset, years		**–0.306**	**–0.326, –0.287**	**–0.100**	**–0.120, –0.080**
Diagnosed by rheumatologist, yes	No	**2.410**	**1.868, 2.952**	**1.163**	**0.710, 1.615**
No. of HCPs seen before diagnosis		**1.696**	**1.520, 1.873**	**1.033**	**0.877, 1.189**
Uveitis	No	**1.580**	**0.996, 2.165**	**1.286**	**0.808, 1.764**
IBD	No	**0.834**	**0.117, 1.550**	–0.043	–0.610, 0.525
Region, Asia	Europe	**–3.511**	**–4.241, –2.781**	**1.003**	**0.334, 1.673**
Region, North America		**1.228**	**0.499, 1.958**	**1.470**	**0.902, 2.039**
Region, Latin America		**–1.792**	**–2.583, –1.000**	0.626	–0.045, 1.297
Region, South Africa		**3.015**	**1.549, 4.481**	**3.356**	**2.170, 4.541**

Dependent variable in all models: diagnostic delay in years. The 95% CIs that do not include 0 and are therefore considered statistically significant are represented in bold text. ^a^The multivariable mode was additionally adjusted for symptom duration at the timepoint of the study inclusion. HCP: health care professional.

## Discussion

The present study surveyed >5000 patients with axSpA from five different regions around the world, comprising a total of 27 countries. The mean diagnostic delay was 7.4 years and was associated with female gender, younger age at symptom onset, being diagnosed by a rheumatologist, greater number of HCPs seen before diagnosis and history of uveitis.

The diagnostic delay of IMAS patients (7.4 years) is slightly higher than that shown by a recent meta-analysis encompassing a total of 64 axSpA studies worldwide (6.7 years) [7], and notably higher than similar cohorts such as ASAS-perSpA (5.8 years) [14]. In addition, we have shown that the regions with the longest diagnostic delay were South Africa (10.8), followed by North America (9.0) and Europe (7.7), while Latin America (5.9) and Asia (4.2) were below the global IMAS average. In this regard, as shown in Supplementary Table S2, available at *Rheumatology* online, in South Africa there may be a relationship between longer diagnostic delay and younger age at symptom onset and longer symptoms duration, while in North America and Europe patients with longer diagnostic delay were associated with more variables including being younger at symptoms onset, longer symptoms duration, female gender, diagnosis by a rheumatologist, a greater number of HPCs seen before diagnosis and presence of uveitis.

The longer diagnostic delay in IMAS patients was associated with female gender. Previous studies have also shown a significant association between female gender and longer diagnostic delay [15, 16]. This may be explained by physician’s bias, as axSpA was long considered to be a predominantly male disease, although more recent studies have shown little difference in prevalence by gender [17, 18]. Furthermore, this greater difficulty in diagnosing women could be due to differences in the symptoms presentation and clinical manifestation, with a greater presence of stiffness in men [19].

IMAS patients with longer diagnostic delay were associated with younger age at symptom onset. These results were also tested in the European IMAS cohort (EMAS) [11] and another study of patients in Germany [20], however this association among a large cohort of patients with axSpA worldwide has not been confirmed until the present study. It is necessary to consider the symptoms and clinical manifestation of the disease in young patients so that they can be referred early to a rheumatologist to confirm their diagnosis.

In addition, being diagnosed by a rheumatologist and a greater number of HCPs seen before diagnosis were associated with a longer diagnostic delay in IMAS patients. It is possible that the association between diagnosis by a rheumatologist and greater diagnostic delay is due to cases that are more difficult to detect by other HCPs, such as primary care physicians, orthopedic specialists or physiotherapists, who do not have the necessary training to diagnose these cases. In this context, it is to be expected that a greater number of HCPs would be seen before diagnosis. In this sense, North America and Europe were the regions with the highest number of HCP visits for diagnosis, while the lowest number of visits was in the Asian region. In general, HLA-B27 test was the most frequently used test for diagnosis of patients in all IMAS regions, more frequently in South Africa and less frequently in Asia. Furthermore, MRI scans and X-rays were performed more frequently in Europe and North America [21]. These difficulties in being diagnosed encountered by patients with axSpA could be the reason for the longer diagnostic delay shown.

Finally, the presence of uveitis in patients with axSpA in the IMAS cohort was associated with longer diagnostic delay. These results are similar to those shown in a study conducted in Europe and Latin America where a significant association was found between the presence of uveitis and a longer diagnostic delay [22]. The presence of uveitis along with chronic back pain could be essential indications to recommend patients to visit a rheumatologist for a possible case of axSpA, thus reducing the diagnostic delay. Waiting >7 years to be diagnosed with axSpA is unacceptable and undoubtedly affects negatively crucial aspects of patients’ lives such as the progression of their disease and their quality of life.

The region factor was introduced to control the effect of the independent variables on diagnostic delay. However, the region factor should be interpreted with caution as the regions are compared with a reference region (Europe) that had the largest sample size and a mean diagnostic delay similar to the overall mean in our study.

Regional variations in diagnostic delays in axSpA may be the result of a combination of health system factors and cultural differences. In this sense, the region with the longest diagnostic delay for IMAS was South Africa, where disparities between public and private health services can result in significant delays in diagnosis and treatment [23]. Furthermore, the limited availability of resources for the diagnosis and treatment of rheumatologic diseases is a significant challenge in many Latin American countries [24]. In North America, socioeconomic disparities can have a significant impact on the time to diagnosis and follow-up of patients with rheumatologic diseases [25]. On the other hand, in Europe, although the diagnostic delay was high, it was lower than that previously mentioned in South Africa, which may be due to the implementation of effective referral protocols in primary care, which is crucial for the early diagnosis of rheumatologic diseases [26].

Finally, although the diagnostic delay in Asia is the lowest of the IMAS regions, it should be noted that it is still over 4 years, which could be due to the preference for traditional medicine over modern medicine in some Asian countries [27].

IMAS is one of the largest surveys of patients with axSpA, including 5557 respondents from 27 countries around the world. Although the diagnostic delay of 5327 IMAS patients from five worldwide regions has recently been published [28], the present manuscript also shows its associated factors in 4595 patients, such as age at symptom onset, symptom duration, gender, diagnosis by a rheumatologist, number of HCPs seen before diagnosis and presence of extra-musculoskeletal manifestations, as well as the region of participants. Therefore, the large sample and the inclusion of other key factors make this a robust and innovative study that provides evidence on how to reduce delay in diagnosis in patients with axSpA. In addition, IMAS is a novel study whose questionnaire was designed with significant contribution from patients.

Despite the above, IMAS has some limitations. First, the survey was based on self-reported data and was not able to confirm the diagnosis of the participants. However, the risk of misdiagnosis in this cohort is not substantially different from any other epidemiological study in axSpA, in which patients with axSpA were recruited by physicians. This is also supported by the fact that the main axSpA-related characteristics in IMAS are similar to those in other published axSpA cohorts [29, 30].

Secondly, information on the presence of extra-musculoskeletal manifestations was gathered at the time of the survey and was not restricted to the period before diagnosis. Finally, there is an overrepresentation of the European region, although to avoid this bias in this analysis the regions were analysed independently.

## Conclusion

In this large sample of patients with axSpA from 27 countries worldwide, the mean diagnostic delay was longer than 7 years. Female gender, younger age at symptom onset, diagnosis by a rheumatologist, a greater number of healthcare professionals seen before diagnosis and the presence of uveitis were associated with longer diagnostic delay. Improved training of healthcare professionals to recognize ‘red flags’ and subsequent rapid referral to a rheumatologist are crucial to reduce diagnostic delay of patients with axSpA.

## Supplementary Material

keae521_Supplementary_Data

## Data Availability

Contact the corresponding author for availability of data.

## References

[1] Braun J , Kiltz U, Baraliakos X. Significance of structural changes in the sacroiliac joints of patients with axial spondyloarthritis detected by MRI related to patients symptoms and functioning. Ann Rheum Dis 2022;81:11–4. 34711586 10.1136/annrheumdis-2021-221406

[2] Siebert S, Sengupta R, Tsoukas A, eds. Axial spondyloarthritis. Oxford University Press, 2016.

[3] Rezvani A , Bodur H, Ataman Ş et al Correlations among enthesitis, clinical, radiographic and quality of life parameters in patients with ankylosing spondylitis. Mod Rheumatol 2014;24:651–6. 24252034 10.3109/14397595.2013.850182

[4] De Winter JJ , Paramarta JE, De Jong HM et al Peripheral disease contributes significantly to the level of disease activity in axial spondyloarthritis. RMD Open 2019;5:e000802. 30713720 10.1136/rmdopen-2018-000802 PMC6340525

[5] de Winter JJ , van Mens LJ, van der Heijde D et al Prevalence of peripheral and extra-articular disease in ankylosing spondylitis versus non-radiographic axial spondyloarthritis: a meta-analysis. Arthritis Res Ther 2016;18:196. 27586785 10.1186/s13075-016-1093-z PMC5009714

[6] de Koning A , Schoones JW, van der Heijde D et al Pathophysiology of axial spondyloarthritis: consensus and controversies. Eur J Clin Invest 2018;48:e12913. 29460306 10.1111/eci.12913

[7] Zhao SS , Pittam B, Harrison NL et al Diagnostic delay in axial spondyloarthritis: a systematic review and meta-analysis. Rheumatology 2021;60:1620–8. 33428758 10.1093/rheumatology/keaa807

[8] Fallahi S , Jamshidi AR. Diagnostic delay in ankylosing spondylitis: related factors and prognostic outcomes. Arch Rheumatol 2016;31:24–30. 29900990 10.5606/ArchRheumatol.2016.5562 PMC5827863

[9] Ryoung Seo M , Lim Baek H, Hwa Yoon H et al Delayed diagnosis is linked to worse outcomes and unfavourable treatment responses in patients with axial spondyloarthritis. 2015;34. 10.1007/s10067-014-2768-y 25185731

[10] Cakar E , Taskaynatan MA, Dincer U et al Work disability in ankylosing spondylitis: differences among working and work-disabled patients. Clin Rheumatol 2009;28:1309–14. 19685294 10.1007/s10067-009-1249-1

[11] Garrido-cumbrera M , Navarro-compán V, Bundy C et al Identifying parameters associated with delayed diagnosis in axial spondyloarthritis : data from the European map of axial spondyloarthritis. Rheumatology 2021;61:1–19. 10.1093/rheumatology/keab369 PMC8824419 33909885

[12] Garrido-Cumbrera M , Poddubnyy D, Sommerfleck F et al International Map of Axial Spondyloarthritis (IMAS): results from the perspective of 5557 patients from 27 countries around the globe. RMD Open 2024;10:e003504. 38851236 10.1136/rmdopen-2023-003504 PMC11163687

[13] Garrido-Cumbrera M , Poddubnyy D, Gossec L et al; EMAS Working Group. The European Map of Axial Spondyloarthritis: capturing the Patient Perspective—an Analysis of 2846 Patients Across 13 Countries. Curr Rheumatol Rep 2019;21:19– 30868287 10.1007/s11926-019-0819-8 PMC6449283

[14] López-Medina C , Molto A, Sieper J et al Prevalence and distribution of peripheral musculoskeletal manifestations in spondyloarthritis including psoriatic arthritis: results of the worldwide, cross-sectional ASAS-PerSpA study. RMD Open 2021;7:1450. 10.1136/rmdopen-2020-001450 PMC7816910 33462157

[15] Jovaní V , Blasco-Blasco M, Ruiz-Cantero MT et al Understanding how the diagnostic delay of spondyloarthritis differs between women and men: a systematic review and metaanalysis. J Rheumatol 2017;44:174–83. 27980009 10.3899/jrheum.160825

[16] Redeker I , Callhoff J, Hoffmann F et al Determinants of diagnostic delay in axial spondyloarthritis: an analysis based on linked claims and patient-reported survey data. Rheumatol 2019;58:1634–8. 10.1093/rheumatology/kez090 30903141

[17] Kennedy LG , Will R, Calin A. Sex ratio in the spondyloarthropathies and its relationship to phenotypic expression, mode of inheritance and age at onset. J Rheumatol 1993;20:1900–4. 8308776

[18] Chimenti MS , Conigliaro P, Navarini L et al Demographic and clinical differences between ankylosing spondylitis and non-radiographic axial spondyloarthritis: results from a multicentre retrospective study in the Lazio region of Italy. Clin Exp Rheumatol 2020;38:88–93. 31140397

[19] Rusman T , van Vollenhoven RF, van der Horst-Bruinsma IE. Gender Differences in Axial Spondyloarthritis: women Are Not So Lucky. Curr Rheumatol Rep 2018;20:35–12. 29754330 10.1007/s11926-018-0744-2 PMC5949138

[20] Redeker I , Callhoff J, Hoffmann F et al Which factors influence the diagnostic delay in patients with axial spondyloarthritis? - ACR meeting abstracts. Am Coll Rheumatol 2018;70(Suppl 9):1839–41. https://acrabstracts.org/abstract/which-factors-influence-the-diagnostic-delay-in-patients-with-axial-spondyloarthritis/ (5 September 2023, date last accessed).

[21] Garrido-Cumbrera M , Poddubnyy D, Sommerfleck F et al Regional differences in diagnosis journey and healthcare utilization: results from the International Map of Axial Spondyloarthritis (IMAS). Rheumatol Ther 2024;11:927–45. 38847994 10.1007/s40744-024-00672-3 PMC11264652

[22] Michelena X , Zhao SS, Marco-Pascual C et al Diagnostic delay is associated with uveitis and inflammatory bowel disease in AS: a study of extra-musculoskeletal manifestations in SpA. Rheumatology (Oxford) 2023;63:430–5. 10.1093/rheumatology/kead225 PMC10836992 37184889

[23] Mayosi BM , Benatar SR. Health and health care in South Africa—20 years after Mandela. N Engl J Med 2 2014;371:1344–53. . 25265493 10.1056/NEJMsr1405012

[24] Pineda C , Caballero-Uribe CV. Challenges and opportunities for diagnosis and treatment of rheumatoid arthritis in Latin America. Clin Rheumatol 2015;34(Suppl 1):S5–7. . 26182887 10.1007/s10067-015-3019-6 PMC4617852

[25] Pincus T , Callahan LF, Burkhauser RV. Most chronic diseases are reported more frequently by individuals with fewer than 12 years of formal education in the age 18-64 United States population. J Chronic Dis 1987;40:865–74. . 3597688 10.1016/0021-9681(87)90186-x

[26] Ramiro S , Nikiphorou E, Sepriano A et al ASAS-EULAR recommendations for the management of axial spondyloarthritis: 2022 update. Ann Rheum Dis 2023;82:19–34. 36270658 10.1136/ard-2022-223296

[27] Chan E , Tan M, Xin J, Sudarsanam S, Johnson DE. Interactions between traditional Chinese medicines and Western therapeutics. Curr Opin Drug Discov Devel 2010;13:50–65. 20047146

[28] Poddubnyy D , Sommerfleck F, Navarro-Compán V et al Regional differences in clinical phenotype of axial spondyloarthritis: results from the International Map of Axial Spondyloarthritis (IMAS). Rheumatology (Oxford) 2023;63:2328–35. 10.1093/rheumatology/kead665 PMC11371368 38128022

[29] López-Medina C , Molto A, Sieper J et al Prevalence and distribution of peripheral musculoskeletal manifestations in spondyloarthritis including psoriatic arthritis: results of the worldwide, cross-sectional ASAS-Perspa study. RMD Open 2021;7:e001450. 33462157 10.1136/rmdopen-2020-001450 PMC7816910

[30] Moltó A , Etcheto A, van der Heijde D et al Prevalence of comorbidities and evaluation of their screening in spondyloarthritis: results of the International cross-sectional ASAS-COMOSPA study. Ann Rheum 2016;75:1016–23. 10.1136/annrheumdis-2015-208174 26489703

