# Supplementary material for: Diagnostic delay in patients from the International Map of Axial Spondyloarthritis: geographic, sociodemographic and disease-related factors
Source: Rheumatology (Oxford). 2024 Sep 25;64(4):1873–9. doi: 10.1093/rheumatology/keae521 (PMC11962975; doi:10.1093/rheumatology/keae521)
Supplement: keae521_Supplementary_Data [file keae521_supplementary_data.docx]

**Supplementary materials**

**Supplementary Table S1.** Description of variables of the study

| **Variables** | **Questionnaire Item** | **Categories/Measures** |
| --- | --- | --- |
| **Socio-demographic** | | |
| Gender | Please specify your gender | Male, female |
| Education level | Please select your level of education completed | No schooling, primary school, high school, university |
| **Diagnosis characteristics** | | |
| Age at onset of first symptoms | Age of onset of first symptoms (pain, inflammation, stiffness) associated with axSpA | In years |
| Age at diagnosis | Age at which you were diagnosed with spondylitis/ spondyloarthritis | In years |
| Diagnostic delay | Calculated as a difference between the age at diagnosis and age at symptom onset | In years |
| Symptom duration | Calculated based on the age at symptom onset | In years |
| HLA-B27 | What was the result of the genetic test (HLA-B27)? | Positive, negative |
| Diagnosed by rheumatologist | Which medical professional made the diagnosis of Spondylitis/ Spondyloarthritis? | Primary care physician, rheumatologist, orthopaedic specialist, physiotherapist, other |
| **Disease extra-musculoskeletal manifestations** | | |
| Uveitis | Please indicate whether you have been diagnosed with any of the following: | Uveitis |
| Psoriasis | Please indicate whether you have been diagnosed with any of the following: | Psoriasis |
| Inflammatory bowel disease | Please indicate whether you have been diagnosed with any of the following: | Inflammatory bowel disease |

**Supplementary Table S2.** Bivariate analysis between sociodemographic and disease-related variables and diagnostic delay by region

| **Variable** | **Diagnostic Delay**  **Mean ± SD or r correlation** | | | | |
| --- | --- | --- | --- | --- | --- |
|  | **Europe** | **North America** | **Latin America** | **Asia** | **South Africa** |
| **Age at symptom onset**  ≤ 18  19-34  35- 51  52-70  **p-value** | 12.9 ± 10.9  7.0 ± 7.7  3.7 ± 4.6  2.0 ± 2.5  **<0.001** | 14.9 ± 14.2  7.9 ± 9.2  4.5 ± 6.2  1.9 ± 3.2  **<0.001** | 14.6 ± 13.2  5.6 ± 6.8  2.8 ± 4.1  0.7 ± 1.7  **<0.001** | 6.6 ± 6.8  4.0 ± 5.1  2.4 ± 3.3  2.0 ± 3.4  **<0.001** | 18.3 ± 11.8  9.7 ± 9.1  3.5 ± 3.6  3.2 ± 1.9  **<0.001** |
| **Gender**  Male  Female  **p-value** | 6.6 ± 7.8  8.5 ± 9.2  **<0.001** | 6.6 ± 9.1  10.4 ± 11.8  **<0.001** | 6.0 ± 8.5  5.9 ± 8.6  0.940 | 4.4 ± 5.5  3.6 ± 5.1  **0.040** | 9.9 ± 10.7  10.9 ± 10.7  0.514 |
| **Education level**  No schooling completed  Primary school  High school  University  **p-value** | 9.3 ± 10.9  7.5 ± 8.8  8.0 ± 8.7  7.5 ± 8.8  0.138 | 2.0 ± 2.8  5.9 ± 5.8  8.6 ± 10.8  9.3 ± 11.2  0.543 | 14.3 ± 17.9  7.4 ± 9.9  7.0 ± 9.7  4.2 ± 6.0  **0.024** | 4.5 ± 4.9  5.9 ± 5.9  4.4 ± 5.4  3.7 ± 5.4  0.066 | -  -  11.2 ± 11.4  9.9 ± 9.3  0.966 |
| **Diagnosed by rheumatologist**  Yes  No  **p-value** | 8.2 ± 9.0  6.1 ± 7.6  **<0.001** | 10.0 ± 11.3  6.8 ± 9.9  **<0.001** | 6.7 ± 8.9  3.7 ± 7.0  **<0.001** | 5.0 ± 6.1  3.3 ± 4.4  **<0.001** | 10.3 ± 10.0  12.2 ± 12.1  0.523 |
| **No. of HCPs seen before diagnosis**  0  1-2  3 or more  **p-value** | 4.4 ± 6.0  6.1 ± 7.9  9.8 ± 9.3  **<0.001** | 2.3 ± 4.0  6.1 ± 9.3  12.0 ± 11.9  **<0.001** | 1.5 ± 2.1  5.0 ± 8.3  7.4 ± 8.8  **<0.001** | 6.9 ± 8.2  4.0 ± 5.5  4.4 ± 4.2  **0.030** | 11.0 ± 14.7  9.7 ± 10.1  13.2 ± 11.1  0.118 |
| **HLA-B27**  Positive  Negative  **p-value** | 8.5 ± 8.5  8.8 ± 9.5  0.406 | 9.6 ± 10.8  11.0 ± 12.0  0.243 | 6.4 ± 8.9  6.1 ± 8.0  0.455 | 4.7 ± 5.2  5.5 ± 6.7  0.653 | 10.8 ± 11.2  8.9 ± 9.8  0.540 |
| **Uveitis**  Yes  No  **p-value** | 8.6 ± 7.7  7.7 ± 8.7  **<0.001** | 10.6 ± 11.7  8.6 ± 10.6  **0.015** | 8.5 ± 10.1  4.6 ± 6.9  **<0.001** | 5.7 ± 5.1  3.5 ± 4.9  **<0.001** | 10.7 ± 11.7  10.3 ± 10.2  0.734 |
| **Psoriasis**  Yes  No  **p-value** | 9.3 ± 10.4  7.6 ± 8.8  **0.041** | 10.2 ± 14.1  9.1 ± 11.5  0.903 | 5.9 ± 8.9  5.3 ± 7.9  0.864 | 4.6 ± 4.8  5.1 ± 5.1  0.701 | 15.7 ± 12.4  10.1 ± 10.1  0.052 |
| **Inflammatory bowel disease**  Yes  No  **p-value** | 8.2 ± 9.0  7.8 ± 8.7  0.473 | 9.6 ± 12.1  9.2 ± 10.9  0.574 | 7.0 ± 9.5  5.3 ± 7.9  0.101 | 5.9 ± 6.2  3.6 ± 4.6  **<0.001** | 12.5 ± 10.2  10.1 ± 10.6  0.091 |

P-values less than 0.05 considered statistically significant are represented in bold text.
